# The Effectiveness and Retention Rate of Iguratimod in Japanese Rheumatoid Arthritis Patients with/without Methotrexate in Daily Medical Care

**DOI:** 10.3390/life10110261

**Published:** 2020-10-29

**Authors:** Asuka Inoue, Yuji Nozaki, Yasuaki Hirooka, Koji Kinoshita, Yasutaka Chiba, Masanori Funauchi, Itaru Matsumura

**Affiliations:** 1 Department of Rheumatology, Kindai University Nara Hospital, Nara 630-0293, Japan; asuka@med.kindai.ac.jp (A.I.); nagare@med.kindai.ac.jp (Y.H.); 2 Department of Hematology and Rheumatology, Kindai University School of Medicine, Osaka 589-8511, Japan; kkino@med.kindai.ac.jp (K.K.); mn-funa@med.kindai.ac.jp (M.F.); imatsumura@med.kindai.ac.jp (I.M.); 3 Department of Clinical Research Center, Kindai University School of Medicine, Osaka 589-8511, Japan; chibay@med.kindai.ac.jp

**Keywords:** iguratimod, methotrexate, rheumatoid arthritis, clinical response, retention rate

## Abstract

(1) Background: We evaluated the clinical response of iguratimod (IGU) in patients with rheumatoid arthritis (RA) being treated with or without methotrexate (MTX) over 54 weeks. (2) Methods: 106 patients with RA undergoing IGU were retrospectively observed. RA patients were divided into those treated with MTX+IGU (*n* = 35) and those treated with IGU (*n* = 71). The primary endpoint was the clinical response of the Disease Activity Score assessing 28 joints with C-reactive protein (DAS28-CRP) differences in the changes from baseline to 54 weeks between MTX+IGU and IGU groups. Secondary endpoints, such as the clinical response, retention rate, and safety, were evaluated. (3) Results: The DAS28-CRP difference in the changes between the two groups were −0.2. DAS28-CRP were significantly reduced from the baseline in the MTX+IGU and IGU groups (−1.43 and −1.20 from baseline, respectively). The retention rates were 71.4% in the MTX+IGU groups and 59.2% in the IGU groups (*p* = 0.16). Adverse events were observed in a total of 6 (17.1%) MTX+IGU patients and 20 (28.2%) IGU patients (*p* = 0.21). (4) Conclusions: IGU therapy may be a useful treatment option for patients who cannot be treated with MTX.

## 1. Introduction

Rheumatoid arthritis (RA) is a well-known autoimmune disease which causes arthritis and is characterized by destruction of cartilage, bone, and tendon. Methotrexate (MTX) remains the anchor drug for the treatment of RA. MTX has contributed to improvement of various clinical symptoms, as well as disease control [1]. In 2016, the European League Against Rheumatism (EULAR) recommended that MTX should be a part of the first-line treatment strategy for the management of RA [2]. For RA patients who show an inadequate response to MTX (MTX-IR), the add-on of another conventional synthetic disease-modifying antirheumatic drug (csDMARD) is an appropriate treatment option [3]. Therefore, a combination therapy using csDMARD may be effective for the patients with MTX intolerance or an inadequate response to initial MTX therapy.

Iguratimod (IGU) is a csDMARD that has been prescribed in daily medical practice for RA patients in Japan since 2012. The therapeutic effects of IGU are derived from its action of suppressing production of inflammatory cytokines, such as tumor necrosis factor alpha (TNFα), interleukin (IL)-1β, IL-6, IL-8, and IL-17 [4,5,6,7], suppression of immunoglobulin production [8], inhibition of the activity of NF-ĸB (nuclear factor kappa-light-chain-enhancer of activated B cells), or direct action on B cells [6,9]. Several studies have reported that IGU improves the clinical symptoms of RA patients [10]. The effectiveness of IGU in RA patients was superior to that of a placebo and not inferior to that of salazosulfapyridine (SASP) [10]. We previously reported that IGU has equivalent therapeutic effects in disease activity compared to SASP with better retention rate and corticosteroid dose-sparing effects [11]. Furthermore, the addition of IGU to MTX has been shown to be effective in RA patients with an inadequate response to MTX monotherapy [12,13]. Especially in elderly patients, there are some cases in which it is difficult to continue or increase the MTX dose due to liver or kidney dysfunction. Therefore, IGU may be an effective treatment option for those who are intolerant to an effective dose of MTX; switching to or adding IGU may also be an effective treatment for RA patients who do not respond adequately to MTX. However, the evidence of the effectiveness of IGU and MTX combinations has been insufficient. In this study, we retrospectively analyzed the effectiveness, retention rate, and safety of IGU with or without MTX in the daily medical care of patients with RA.

## 2. Materials and Methods

### 2.1. Patients

This study was conducted at a single center, Kindai University School of Medicine in Osaka, Japan. The study included 106 Japanese patients diagnosed with RA who were treated with IGU during the period from 2014 to 2017. RA was diagnosed according to the 2010 American College of Rheumatology (ACR) criteria and the EULAR classification criteria [14,15]. Patients were excluded if they had been treated with biological DMARDs (bDMARDs). Patients with a connective tissue disease other than RA and patients previously treated with IGU were also excluded from the study. All data were retrospectively collected from clinical and laboratory data from electronic medical records. We divided the patients into two groups—those treated with both IGU and MTX (MTX+IGU group; *n* = 35), and those treated with IGU without MTX (IGU group; *n* = 71), while patients were permitted to continue the other csDMARDs which had been already taken before this study. The observation period of this study was 54 weeks. The baseline data was obtained when the IGU treatment was initiated. Patients who had been treated with MTX before they started IGU therapy were assigned to the MTX+IGU group. To evaluate the appropriate therapeutic effect of MTX, in the MTX+IGU group, patients discontinued MTX within 24 weeks after addition of IGU were excluded. Patients who were not treated with MTX during the observation period were assigned to the IGU group. In the IGU group, patients who started MTX after initiating IGU therapy were excluded. Concomitant oral glucocorticoids (stable dose ≤7.5 mg of prednisolone (PSL)), SASP, bucillamine (BUC), or tacrolimus (TAC)) were permitted in both groups. The doses of PSL, SASP, BUC, and TAC could be adjusted at the discretion of the physician in charge.

IGU was orally administered to the RA patients in both groups at the dose of 25 mg for the first 4 weeks and 50 mg thereafter. MTX was orally administered to the MTX+IGU group at a dose of 2–16 mg/week, and the dosages were determined also at the physician’s discretion.

### 2.2. Compliance with Ethical Standards

This study was conducted according to the principles expressed in the Helsinki Declaration of 1983 and was approved by the Research Ethics Committee of Kindai University School of Medicine. This study was registered at the Research Ethics Committee of Kindai University School of Medicine (registration no. 30-061) and University Hospital Medical Information Network (ID; UMIN000041767, no. R000047673). Written informed consent for their data to be used was obtained from all patients.

### 2.3. Demographic Characteristics and the Assessment of Clinical and Laboratory Data

The 106 patients’ characteristics were recorded at baseline including the age, sex, disease duration, current therapy, and the anti-cyclic citrullinated peptide antibody (ACPA) titer. At baseline, 24 weeks, and 54 weeks, the recorded clinical data included the 28 swollen and tender joint counts (SJC and TJC) and a patient visual analogue scale (Pt-VAS) in which 0  =  best and 100  =  worst. The following laboratory data were assessed at baseline, 24 weeks, and 54 weeks: C-reactive protein (CRP), the erythrocyte sedimentation rate (ESR), matrix metalloproteinase-3 (MMP-3), and rheumatoid factor (RF).

### 2.4. Assessments of Clinical and Laboratory Data

The effectiveness parameters included the DAS28-CRP, TJC, SJC, and Pt-VAS, and the laboratory parameters of CRP (mg/dL), ESR (mm/h), MMP-3 (ng/mL), and RF (IU/mL). The differences in the changes from baseline in absolute value were measured and compared between the MTX+IGU and IGU groups.

### 2.5. Definitions of Disease Activity

With respect to the DAS28-CRP disease activity categories [16], we divided the patients into those at remission (DAS28-CRP < 2.3) and those in non-remission as follows. Low: 2.3 ≤ DAS28-CRP < 2.7, Moderate: DAS28-CRP 2.7-4.1, High: DAS28-CRP > 4.1.

### 2.6. Retention Rate

We analyzed the IGU retention of the MTX+IGU and IGU groups by using the Kaplan–Meier method for up to 48 months from 2014 to 2018. A log-rank test was performed for the comparison of the MTX+IGU and IGU groups. For RA treatment, the patient’s age and the presence of interstitial pneumonia are important factors in deciding whether to choose MTX; therefore, to reduce the bias associated with age and the presence of interstitial pneumonia, we adjusted the age of both groups and excluded patients with interstitial pneumonia.

### 2.7. Safety and Reasons for Discontinuation

The treating physicians evaluated the physical examination findings, clinical symptoms, laboratory results, and chest radiographs. A physical examination and the laboratory tests were evaluated at each patient visit during the treatment period. Chest radiographs were taken when respiratory symptoms were present. If IGU was discontinued, the physician in charge recorded the reason for discontinuation and made any adjustments to the treatment based on his/her assessment.

### 2.8. Statistical Analyses of Patients

Statistical analyses were performed using GraphPad prism software (GraphPad Software, San Diego, CA, USA) and SAS 9.4 (SAS Institute Inc., Cary, NC, USA). The primary endpoint was the clinical response of the DAS28-CRP differences in the changes from baseline to 54 weeks between MTX+IGU and IGU groups. The secondary endpoints were effectiveness between the MTX+IGU and IGU groups that were made on clinical responses (DAS28-CRP, VAS, TJC, SJC, CRP, ESR, MMP-3, and RF titers), retention rate, and safety. For the clinical responses, the differences of delta values between two treatment groups were derived, where the delta values were defined as the differences between measurements at 24 weeks and baseline and between 54 weeks and baseline. For the time-to-retention, Kaplan–Meier curves were estimated. In addition, as age was considered as a confounder in this retrospective study, for both clinical responses and time-to-retention, statistical analyses to adjust for age were performed using the inverse-probability-weighting (IPW) method [17], where the weight was yielded based on the propensity score. The reasons for IGU discontinuation and the side effects in each group were investigated by χ² test. Summary statistics of the mean ± standard deviation (SD) or the median and interquartile range (IQR) are presented for continuous variables as appropriate. Categorical variables are presented as percentages. Comparisons between independent means were analyzed using the Mann–Whitney U-test. Relationships between categorical variables were evaluated by the χ² test. *p*-values < 0.05 were considered significant. The missing data were excluded from the analysis without supplementing the data with estimated or calculated values.

## 3. Results

### 3.1. Patient Characteristics

The baseline characteristics of the IGU group and MTX+IGU group are summarized in Table 1. The patients in the IGU group were significantly older than those in the MTX+IGU group (64.8 vs. 56.9 years, respectively; *p* = 0.02). There were no significant differences between the two groups in the disease duration of RA, clinical disease activity (DAS28-CRP, TJC, SJC, and Pt-VAS), or the positive percentage and titers of RF or ACPA. In the MTX+IGU group, the mean dose of MTX was 7.8 ± 0.5 mg/week. There were no significant differences between the patient groups in the percentage of users of PSL, SASP, BUC, or TAC.

### 3.2. Clinical Effectiveness

**Figure 1:**
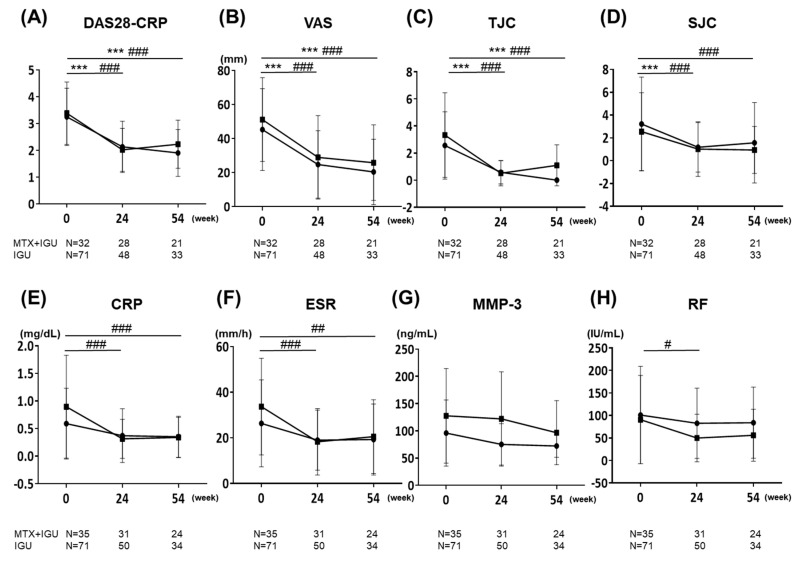
The clinical courses of physical findings and laboratory data (DAS28-CRP, VAS, TJC, SJC, CRP, ESR, MMP-3, and RF) in the IGU group and MTX+IGU group for 54 weeks. (**A**) DAS28-CRP. (**B**) VAS. (**C**) TJC. (**D**) SJC. (**E**) CRP. (**F**) ESR. (**G**) MMP-3. (**H**) RF. */#: *p* < 0.05, **/##: *p* < 0.01, ***/###: *p* < 0.001 vs. the parameters at baseline: *MTX+IGU group, #IGU group. DAS28-CRP: Disease Activity Score assessing 28 joints with C-reactive protein, IGU: iguratimod, MTX: methotrexate, Pt-VAS: visual analogue scale, TJC: tender joint count, SJC: swollen joint count, CRP: C-reactive protein, ESR: erythrocyte sedimentation rate, MMP-3: matrix metalloproteinase-3, RF: rheumatoid factor.

We analyzed the changes from the patients’ baseline values in the clinical assessment. Figure 1 shows the disease activity, clinical response, and laboratory data at 24 and 54 weeks, i.e., the DAS28-CRP, VAS, TJC, SJC, CRP, ESR, MMP-3, and RF changes from baseline. The patients’ DAS28-CRP scores were significantly reduced from baseline in both the MTX+IGU and IGU groups (−1.43 and −1.20 from baseline, respectively). Other parameters were also significantly reduced from baseline in both groups. At both 24 and 54 weeks, the CRP values in both treatment groups were significantly reduced compared to the baseline. At 54 weeks, the MMP-3 values and the RF titer were not significantly decreased from the baseline in both of the treatment groups. Since there are missing data for each covariate, the number of each parameter is presented below the figure.

The differences in the absolute change from baseline in each clinical parameter at 24 and 54 weeks in both groups are listed in Table 2(a). DAS28-CRP differences in the changes from baseline to 54 weeks between MTX+IGU and IGU groups, which was the primary endpoint in this study, was −0.2 (*p* = 0.52) with no significant difference. For other clinical parameters, there were also no significant differences in the amount of change from baseline in the clinical data between the two groups at 24 weeks or at 54 weeks. Since there was a significant difference in age between the two groups at baseline, we performed a statistical adjustment for age, and the results of the amount of change from baseline in the clinical parameters between the two groups after age adjustment are shown in Table 2(b). Even after the age adjustment, there was no significant difference in the clinical parameters between the two groups.

**Figure 2:**
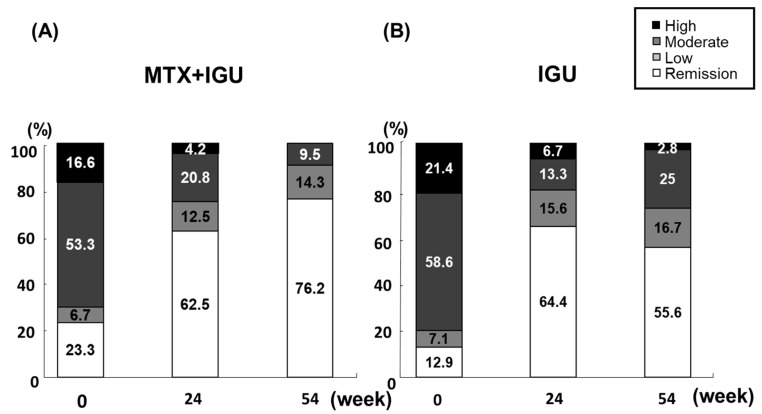
Disease activity in the DAS28-CRP. Time course of the disease activity in DAS28 through 54 weeks following the initiation of RA treatment in the MTX+IGU and IGU groups. (**A**) MTX+IGU group. (**B**) IGU groups. The disease activity and criteria in DAS28-CRP were categorized as follows. The DAS28 criteria: ■ High: 4.1 < DAS28. ■ Moderate: 2.7 ≤ DAS28 ≤ 4.1. ■ Low: 2.3 ≤ DAS28 < 2.7. □ Remission: DAS28 < 2.3.

In the evaluation of remission based on the DAS28 criteria at months 0, 24, and 54, as shown in Figure 2, we found that the proportions of patients achieving remission at these time points were not significantly different between the MTX+IGU and IGU groups: 23.3% vs. 12.9% at baseline, 62.5% vs. 63.0% at 24 weeks, and 76.2% vs. 55.6% at 54 weeks, respectively.

### 3.3. MTX Treatment

In the MTX+IGU group, all patients used MTX in combination until 24 weeks and 6 patients discontinued MTX between 24 and 54 weeks. At baseline, the MTX dosage was 7.8 ± 3.2 mg/week in the MTX+IGU group. At 24 and 54 weeks, the MTX dosages were 7.2 ± 3.0 and 6.1 ± 4.2 mg/week, respectively. The MTX dose did not differ significantly during the 54 weeks of the study period.

### 3.4. Retention Rate

**Figure 3:**
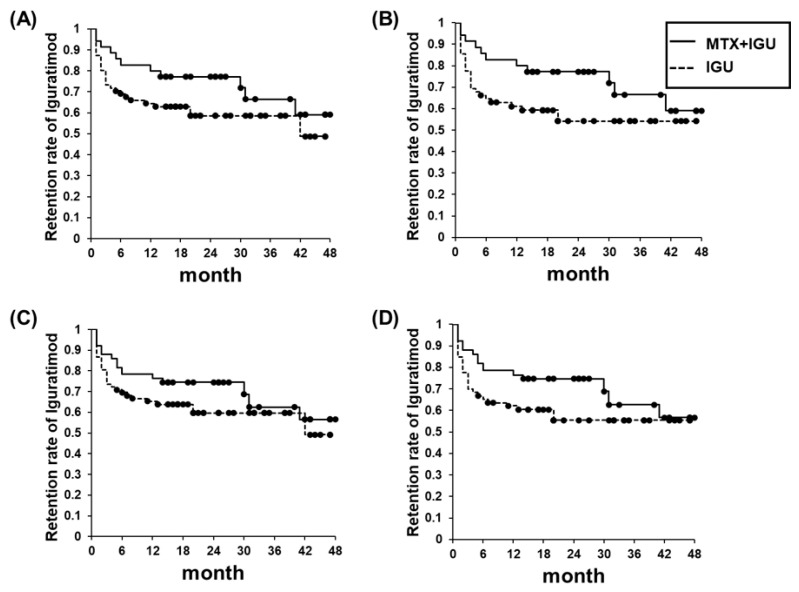
Retention rates of the 106 RA patients for 48 months. Kaplan–Meier curves for the MTX+IGU group (*n* = 35) and IGU group (*n* = 71). (**A**) No age adjustment. (**B**) No age adjustment without patients with interstitial pneumonia (IP). (**C**) With age adjustment. (**D**) With age adjustment without IP.

Figure 3 shows the retention rates of IGU treatment in the MTX+IGU and IGU groups for up to 48 months from 2014 to 2018. The IGU retention rate of all RA patients for the 54 weeks was 63.2% (67/106). The MTX+IGU and IGU groups’ retention rates were 82.9% (29/35) and 66.2% (47/71) at 24 weeks and 71.4% (25/35) and 59.2% (42/71) at 54 weeks, respectively. There were no significant differences in retention rates between the two groups (*p* = 0.16, hazard ratio (HR) 0.62, Figure 3A). Since factors such as age and the presence or absence of interstitial pneumonia may affect the retention rate, we also evaluated the retention rates excluding these factors, and we observed that even when the patients with interstitial pneumonia were excluded, no significant difference was present in the retention rates of the two groups (*p* = 0.11, HR 0.58, Figure 3B). There was no significant difference in the retention rates between the MTX+IGU and IGU groups even after adjusting for age (*p* = 0.34, HR 0.72, Figure 3C; *p* = 0.27, HR 0.68, Figure 3D).

### 3.5. Reasons for Discontinuation; Safety Evaluation

The reasons for IGU discontinuation are shown in Table 3. IGU was discontinued due to adverse events, improved disease activity, inadequate therapeutic effect, patient request, and death from other underlying disease. Adverse events were observed in a total of 6 (17.1%) patients in the MTX+IGU group and 20 (28.2%) patients in the IGU group. There were no significant differences between the two groups in the total number of adverse events (*p* = 0.21). Liver dysfunction was the most common adverse event leading to IGU discontinuation in the IGU group and was noted in 12 patients (16.9%). In the MTX+IGU group, there were no patients whose IGU was discontinued due to liver dysfunction. There was a significant difference in the rate of liver dysfunction between the two groups (*p* = 0.01).

After the age adjustment, similar results were obtained considering the age differences between the groups (odds ratio: 0.99, 95% CI: 0.96–1.04). Among the 12 patients with liver dysfunction in the IGU group, six patients had either aspartate aminotransferase (AST) and/or alanine aminotransferase (ALT) values that were >100 IU/L. In addition, 16.9% of patients in the IGU group had liver dysfunction requiring IGU discontinuation. This hepatic dysfunction was temporary and was almost eliminated by discontinuing the IGU.

## 4. Discussion

There have been several reports on the therapeutic effects of the combination of IGU and MTX [18,19]. Okamura et al. retrospectively examined patients treated with IGU, and reported the therapeutic effects of IGU with and without MTX [20]. They noted that at 52 weeks, the disease activity was significantly decreased from baseline in both the patients with and without MTX treatment. As a study showing the usefulness of MTX combination in IGU therapy, Ishiguro et al. reported the combination of MTX as a predictor of good response in a post-hoc analysis of the post-marketing surveillance of IGU [21]. However, in these previous reports, the results were not analyzed statistically between the groups with and without MTX. Thus, this study evaluated the effectiveness of IGU with or without MTX in a single hospital in Osaka, Japan, in 35 patients in MTX+IGU and 71 as IGU therapy. The study found that at 54 weeks, 71.4% in the MTX+IGU group and 59.2% in the IGU group were still under treatment, with 76.2% and 55.6% in remission.

The results of our present retrospective analyses of 106 patients with RA revealed that both the IGU and MTX+IGU treatment groups showed a significant decrease in DAS28-CRP at 54 weeks compared to the baseline, with no significant difference between the two groups. Differences in baseline disease activity may be related to population selection bias, but there was no significant difference in disease activity between the two groups. Recent studies have reported that low baseline DAS28-CRP levels are predictors of remission and low disease activity achievement in IGU treatment [20,22]. These studies included many patients with high disease activity, while the baseline DAS28-CRP scores in our present patients were relatively lower. The low baseline DAS28-CRP scores in the present investigation might be one reason why there was no difference in effectiveness between the MTX+IGU and IGU groups. In RA patients with a low baseline DAS28-CRP score, the therapeutic effect of IGU may be fully expected even in patients who cannot be treated with MTX.

In a post-marketing study, Mimori et al. reported that the retention rate of IGU-treated patients was 56.3% at 52 weeks [23]. Okamura et al. observed that the retention rates at 52 weeks were 75.0% and 23.5% in RA patients with and without MTX treatment, respectively [20]. They did not statistically make comparisons with and without MTX groups. In the present study, the total retention rate for both groups at 54 weeks was 63.2%, and the retention rates were 71.4% and 59.2% in the MTX+IGU and IGU groups, respectively. The disparities in the retention rates of previous studies may be due to differences in patient baseline characteristics, such as the disease activity, disease durations, stage, comorbidities, and treatment-related factors (dose, concomitant medications).

It has been reported that liver dysfunction was an adverse reaction characteristic of IGU therapy [23,24,25]. Mimori et al. reported that the incidence of liver dysfunction was higher in their IGU monotherapy group (13%) than in the IGU+MTX group (6.2%) [24]. As was found in our study, although the reasons why liver dysfunction is more common in patients not treated with MTX are unknown, it is important to note that IGU therapy requires the monitoring of liver enzymes regardless of MTX administration and the patient’s age.

Herein, the MTX+IGU group included patients with MTX-IR, for whom it is difficult to reduce PSL and csDMARDs doses or discontinue these agents, and/or difficult to increase the MTX dose sufficiently. The present IGU group included patients who could not be treated with MTX, such as those with interstitial pneumonia (IP) or renal dysfunction.

Our results demonstrated a good therapeutic effect and a good retention rate of IGU with or without MTX treatment. However, there was a significant difference in baseline age between the IGU and MTX+IGU groups. In general, MTX may not be used to treat elderly patients due to their reduced renal function, which may explain why the present IGU patients were older. Similar results were obtained in our IGU and MTX+IGU groups after the adjustment for age.

Our study has some limitations. First, the lack of randomization and blinding may have resulted in bias due to indication and selection of the patients. In this study, the adjustment of medications, including csDMARDs and PSL, was determined by each physician, and there was no uniformed protocol. However, gender, disease duration, seropositivity, and disease activity may also be variables that affect the efficacy and retention of RA drugs, and our adjustment for confounding factors may be inadequate. Second, the study population was rather small (*n* = 106), which may lead to low statistical power and accuracy of the analysis. In addition, there was a difference in the number of patients in the MTX+IGU and IGU groups. Third, there was a lot of missing data in our study. An increase in missing data leads to a decrease in sample size and analysis accuracy, resulting in bias. Missing data arose due to discontinuation of therapeutic drugs, and the decision was left to each physician. Since this study is a retrospective study, there were no preset treatment discontinuation criteria. Discontinuation of treatment at the discretion of each physician could also lead to bias. Thus, a randomized control trial is indispensable to establish the effects of IGU treatment with and without MTX.

## 5. Conclusions

IGU therapy was not inferior to the clinical efficacy and retention rate of MTX+IGU therapy. IGU therapy may be a useful treatment option for patients who cannot be treated with MTX.

## Figures and Tables

**Table 1:** The baseline characteristics of the rheumatoid arthritis (RA) patients.

	MTX+IGU Group *n* = 35	IGU Group *n* = 71	*p*-Value
Age	56.9 ± 16.5	64.8 ± 13.5	0.02 *
Disease duration, months	65.4 ± 70.6	58.9 ± 69.8	0.30
Dose of MTX, mg/week	7.8 ± 3.2	-	-
PSL use at baseline, %	14.3	22.5	0.32
SASP use at baseline, %	45.7	32.4	0.18
BUC use at baseline, %	5.7	7	0.80
TAC use at baseline, %	0	8.5	0.17
DAS28-CRP	3.3 ± 1.1	3.4 ± 1.2	0.62
Pt VAS, 0–100 mm	45.2 ± 24.0	51.1 ± 24.6	0.24
TJC	2.6 ± 2.5	3.3 ± 3.1	0.21
SJC	2.9 ± 3.7	1.8 ± 1.9	0.80
CRP, mg/dL	0.6 ± 0.6	0.8 ± 0.8	0.21
ESR, mm/h	26.4 ± 19.1	33.7 ± 21.2	0.10
MMP-3, ng/mL	96.0 ± 60.6	127.6 ± 86.9	0.15
RF positive, %, titer, IU/mL	68.6, 100.9 ± 108.3	77.5, 90.7 ± 98.1	0.32, 0.85
ACPA positive, %, titer, U/mL	71.4, 176.6 ± 178.6	74.6, 91.5 ± 86.8	0.13, 0.17

* *p* < 0.05. Values are mean ± SD, ACPA: the anti-cyclic citrullinated peptide antibody, BUC: bucillamine, CRP: C-reactive protein, DAS: Disease Activity Score, ESR: erythrocyte sedimentation rate, IGU: iguratimod, MMP-3: matrix metalloproteinase-3, MTX: methotrexate, PSL: prednisolone, RF: rheumatoid factor, SASP: salazosulfapyridine, SJC: swollen joint count, TAC: tacrolimus, TJC: tender joint count, VAS: visual analogue scale.

**Table 2:** Difference in the changes from baseline to 24 and 54 weeks between MTX+IGU and IGU groups.

	Difference in the Changes from Baseline to 24 Weeks (95% CI)	*p*-Value	Difference in the Changes from Baseline to 54 Weeks (95% CI)	*p*-Value
**(a) no Age Adjustment**				
DAS28-CRP	0.2 (−0.4–0.7)	0.58	−0.2 (−0.9–0.5)	0.52
Pt VAS, 0–100 mm	5.8 (−8.2–19.9)	0.41	1.0 (−13.0–15.0)	0.88
TJC	0.4 (−1.1–1.9)	0.58	0.4 (−1.4–2.1)	0.67
SJC	0.0 (−1.6–1.6)	0.97	0.9 (−1.3–3.0)	0.41
CRP, mg/dL	0.0 (−1.0–0.9)	0.96	−0.3 (−1.2–0.7)	0.59
ESR, mm/h	2.7 (−4.5–9.9)	0.45	−0.3 (−8.7–8.0)	0.94
MMP-3, ng/mL	−73.7 (−261.0–113.5)	0.42	−103.1 (−310.0–103.7)	0.52
RF, IU/mL	22.6 (−26.9–72.1)	0.36	−0.2 (−0.9–0.5)	0.88
**(b) with Age Adjustment**				
DAS28-CRP	0.1 (−0.4–0.7)	0.60	0.3 (−1.0–0.32)	0.31
Pt VAS, 0–100 mm	6.3 (−6.8–19.5)	0.34	−0.1 (−13.9–13.6)	0.98
TJC	0.4 (−1.0–1.8)	0.59	0.2 (−1.5–1.9)	0.78
SJC	−0.2 (−1.8–1.3)	0.76	0.5 (−1.6–2.5)	0.64
CRP, mg/dL	−0.2 (−1.2–0.8)	0.68	−0.5 (−1.5–0.5)	0.34
ESR, mm/h	0.7 (−6.7–8.0)	0.86	−2.7 (−11.2–5.9)	0.53
MMP-3, ng/mL	−50.0 (−243.4–143.4)	0.60	−78.1 (−288.4–132.2)	0.45
RF, IU/mL	15.6 (−33.3–64.5)	0.52	42.0 (−45.3–129.4)	0.34

Change in each clinical data value (DAS28-CRP, VAS, TJC, SJC, CRP, ESR, MMP-3, and RF levels) from baseline were compared between the MTX+IGU group and the IGU group at 24 and 54 weeks; {ΔMTX+IGU group (24 weeks–baseline)}–{ΔIGU group (24 weeks–baseline)}, {ΔMTX+IGU group (54 weeks–baseline)}–{ΔIGU group (54 weeks–baseline)}. CI: confidence interval, CRP: C-reactive protein, DAS: Disease Activity Score, ESR: erythrocyte sedimentation rate, IGU: iguratimod, MMP-3: matrix metalloproteinase-3, MTX: methotrexate, RF: rheumatoid factor, SJC: swollen joint count, TJC: tender joint count, VAS: visual analogue scale.

**Table 3:** Reasons for IGU discontinuation.

Reasons for IGU Discontinuation	MTX+IGU Group *n* = 35	IGU Group *n* = 71	*p*-Value
SAE	6	20	0.21
Liver dysfunction	0	12	0.01 *
Eruption	2	2	0.46
Gastritis	0	1	0.48
Nausea	1	0	0.15
Diarrhea	0	1	0.48
Paresthesia	1	1	0.61
Pneumonia	0	1	0.48
Hypoglobulinemia	1	0	0.15
Thrombocytopenia	0	1	0.48
Lymphadenopathy	1	1	0.61
Others	4	9	-
No improvement	3	6	0.98
Improvement	1	1	0.61
Own interruption	0	1	0.48
Death (due to other underlying diseases)	0	1	0.48

* *p* < 0.05, MTX+IGU group vs. IGU group. IGU: iguratimod, MTX: methotrexate, SAE: severe adverse event.
